# Interactions between dendritic cells and CD4^+ ^T cells during *Plasmodium *infection

**DOI:** 10.1186/1475-2875-7-88

**Published:** 2008-05-21

**Authors:** Carlos Ocaña-Morgner, Kurt A Wong, Ana Rodriguez

**Affiliations:** 1 New York University School of Medicine, Department of Medical Parasitology, 341 E 25th street, New York, NY 10010, USA; 2 Institute of Physiological Chemistry, Medical School, MTZ, Dresden University of Technology, Fiedlerstr. 42, 01307 Dresden, Germany

## Abstract

**Background:**

During infection, dendritic cells (DCs) encounter pathogenic microorganisms that can modulate their function and shape the T cell responses generated. During the process of T cell activation, DCs establish strong, long-lasting interactions with naïve T cells.

**Methods:**

Using a mouse malaria model, the interactions of DCs and naïve CD4^+ ^T cells have been analysed.

**Results:**

DCs, either incubated *in vitro *with infected erythrocytes or isolated from infected mice, are able to present exogenous antigens by MHC-II, but are not able to establish prolonged effective interactions with naïve CD4^+ ^T cells and do not induce T cell activation. It was also found that effective T cell activation of naïve CD4^+ ^T cells is impaired during late *Plasmodium yoelii *infection.

**Conclusion:**

These data may provide a mechanism for the lack of effective adaptive immune responses induced by the Plasmodium parasite.

## Background

Dendritic cells (DCs) are antigen-presenting cells (APC) that play a central role in both innate and adaptive immune responses. To initiate T cell-dependent immune responses to microbial infections, DCs phagocytose antigens in peripheral tissues and migrate to the draining lymph nodes, where they interact with antigen-specific T cells. Maturation of DCs, involving up-regulation of the major histocompatibility complex (MHC) and peptide complexes and the costimulatory molecules at the surface, is required to efficiently prime naïve T cells [1]. Upon maturation, DCs reorganize their actin cytoskeleton, projecting long and motile membrane extensions, called dendrites. The initial encounters between antigen-presenting DCs and specific naïve T cells are characterized by the directional projection of abundant membrane extensions from the DC toward the naïve T cell, followed by entrapping of the T cell within a complex net of membrane extensions [2].

The activation of T cells by DCs during *Plasmodium *infection has been previously studied. Although different effects have been described depending on the parasite strain used, time after infection or subpopulation of DC analysed, a number of reports found defective activation of T cells [3]. These findings may be related with the low parasite-specific T cell responses induced by human malaria infections [4,5].

This report shows that DCs from *Plasmodium yoelii*-infected mice are able to present antigens associated with MHC-II, but do not establish strong interactions with naïve CD4^+ ^T cells. Accordingly, it was also found that activation of naïve CD4^+ ^T cells is inhibited during late malaria infections.

## Methods

### Parasites and mice

*Plasmodium yoelii *(non-lethal parasite line 17 XNL) sporozoites were obtained from dissection of infected *Anopheles stephensi *mosquito salivary glands. BALB/c (haplotype *H-2K*^*d*^), C57BL/6 (haplotype *H-2K*^*b*^) and Swiss Webster mice were purchased from Taconic (Germantown, NY). DO11.10 transgenic mice expressing a TCR specific for an epitope from chicken ovalbumin (OVA) on CD4^+ ^T cells were purchased from Jackson Laboratories (Bar Harbor, ME).

### Erythrocytes isolation and mice infection with *P. yoelii*-infected erythrocytes

*Plasmodium yoelii*-infected erythrocytes were obtained from infected Swiss Webster mice with >25% parasitemia. *P. yoelii*-infected erythrocytes were washed three times with PBS and separated from white blood cells by centrifugation at 2,000 *g*. Uninfected erythrocytes were obtained from non-infected mice in the same way. To induce blood-stage infection, 4 × 10^6 ^*P. yoelii*-infected erythrocytes were injected i.v. into each mouse. Parasitemia was measured on days 2, 5, 8, 10, 13 and 15 with values of 4, 12, 25, 45, 20 and 11%, respectively.

### Preparation of DCs from mouse bone marrow and incubation with *P. yoelii*-infected erythrocytes

Primary cultures of immature DCs from BALB/c mice were obtained by differentiation of bone marrow-derived precursors as described [6]. This preparation yields >90% of DCs expressing the distinctive DC surface marker CD11c, as well as MHC class I and II molecules [7]. *Plasmodium yoelii*-infected erythrocytes from infected mice were separated into schizont and non-schizont forms by centrifugation in Accudenz (Accurate Chemical & Scientific Corp, Wertbury, NY) density gradient solution. Uninfected erythrocytes were treated in the same way. DCs (10^6 ^cells/ml) were incubated for 24 h with uninfected or *P. yoelii*-infected erythrocytes at a 1:30 ratio (DCs:schizonts). Then, co-cultures were incubated with erythrocyte lysis buffer (155 mM NH_4_Cl, 1 mM KHCO_3_, 0.1 mM Na_2_EDTA) for 5 min. When indicated, maturation of DCs was induced after 24 h of incubation with erythrocytes by addition *Salmonella enterica *LPS (1 μg/ml) (Sigma, St. Louis, MO).

### Isolation of DCs from mice

CD11c^+ ^DCs were purified from the spleens of groups of three malaria-infected at different times during the infection and non-infected mice using anti-CD11c antibodies bound to magnetic beads (Miltenyi Biotec, Auburn, CA). The isolated cell population is >85% CD11c positive.

### Monoclonal antibodies and flow cytometry

PE anti-CD4 (L3T4), FITC anti CD69 (H1.2F3), PE anti-DO11.10 clonotypic TCR (KJ1-26) from BD Biosciences. Cell preparations were analysed on a FACSCalibur ^® ^(Becton Dickinson). Results are shown after subtraction of the background with only the secondary antibody.

### Antigen Processing and Presentation

Using specific antibodies: Bone marrow-derived DCs pre-incubated for 24 h with uninfected or *P. yoelii*-infected erythrocytes were incubated with 5 mg/ml OVA (Sigma) or 50 μg/ml LACK for 5 h. After incubation, cells were washed and incubated with LPS (1 μg/ml) (Sigma) for 16 h. Expression of MHC-peptides complexes was detected by immuno-fluorescence with specific antibodies and analyzed using FACSCalibur^® ^(Becton Dickinson). Alexa 647 conjugated antibody 25-D1.16 specific for the OVA peptide 257–264/*K*^*b *^complex was provided by Dr. Jon Yewdell (NIH, Bethesda, MD) [Porgador, 1997 #144]. The immunodominant *Leishmania *LACK protein and antibody 2C44 (Glaichenhaus, manuscript in preparation), which recognizes the LACK 156–173/I-A^d ^complex (LACK sequence FSPSLEHPIVVSGSW) [Malherbe, 2000 #181] was provided by (Nicholas Glaichenhaus, Université de Nice-Sophia Antipolis, Valbonne, France). FITC-Avidin was from Sigma (St. Louis, MO). Using T-cell hybridomas: the same procedure was used as in with FACS except that only 0.5 mg/ml of OVA was used and CD11c^+ ^DCs from groups of three uninfected or *P. yoelii*-infected mice at different times after infection were also analyzed. After LPS addition, cells were fixed with 0.001% glutaraldehyde for presentation of OVA peptide 257–264/*H-2*^*b *^complex or 1% paraformaldehyde for OVA peptide 323–339/I-A^d ^complex. Presentation of peptides-MHC complexes was detected using T-cell hybridomas B3Z (provided by Dr. Ronald Germain, NIH, Bethesda, MD) for OVA epitope 257–264/*H-2*^*b *^complex and DO-11.10 (provided by Dr. John Kappler and Dr. Pippa Marrack, Howard Hughes Medical Institute, Denver, CO) for OVA epitope 323–339/I-A^d ^complex. 2 × 10^5 ^DCs of matching haplotype were mixed with 10^5 ^T-cell hybridomas in 200 μl of media/well in a 96-well plate. Supernatant was collected after 24 h and IL-2 secretion was measured by ELISA (BD Biosciences).

### DC-T cell contact

Bone marrow derived DCs previously incubated with uninfected or *P. yoelii*-infected erythrocytes (see above) and CD11c^+ ^DCs from the spleens of groups of three blood stage-infected mice were incubated or not with 10 μg/ml of LPS for 20 h before incubation with OVA peptide 323–339 (ISQAVHAAHAEINEAGR) (Biopeptide Company, San Diego, CA) at 37°C for 1 h. DC-T cell contact was analysed by immunoflurescence, time-lapse video microscopy and FACS as previously described [8]. For immunofluorescence, DCs were washed twice with PBS and immobilized on poly-L-lysine-coated coverslips for 5 min at room temperature (10^5 ^cells/coverslip). PBS was then removed and replaced with complete medium and the coverslips were incubated for 1 h at 37°C. For DC-T cell conjugate formation, 10^5 ^naïve DO11.10 T cells isolated from transgenic mice were loaded with CFSE (0.5 μM) and added to DCs and incubated at 37°C. Incubation was stopped after 30 min and coverslips were washed five times with PBS and fixed with 1% paraformaldehyde for 10 min. F-actin was stained using rhodamine-conjugated phalloidin (Molecular Probes) (1:500 in blocking-permeabilization solution). F-actin is used to differentiate DCs from T cells, as the later have much lower levels of F-actin [8]. DC-T cell contact was reported as the number of 'engulfed' T cell per 100 DCs in each each coverslip. For time-lapse video microscopy, chambers mounted on a coverglass (Nalge Nunc International) coated with 10^5 ^DCs were placed on the microscope at 37°C. One minute after addition of 10^5 ^naïve DO11.10 T cells images were collected every 10 seconds for 20 min. Images were acquired using a 60× oil immersion objective and a Hamamatsu digital camera (Universal Imaging). For FACS, pre-stained naïve DO11.10 T cells with 0.5 μM CFSE and DCs with 1 μM of CellTracker™ Orange (Molecular Probes) were mixed (1:1), spun for 3 min at 500 rpm (4°C) and incubated at 37°C for 30 min. Contact was stopped by transferring the tubes to ice. Analysis by FACS was done immediately after and the results are expressed as percentage of green-red events to total T cells.

### Stimulation of naïve DO11.10 CD4^+ ^T cells

Immature and mature bone marrow derived DCs previously incubated with uninfected or *P. yoelii*-erythrocytes and CD11c^+ ^DCs from the spleen of groups of three blood stage-infected mice at different time points were incubated or not with 10 μg/ml of LPS for 20 h before incubation with 10 μg/ml of OVA peptide 323–339 as above. After washing, 2 × 10^5 ^DCs were incubated with 2 × 10^5 ^naïve CD4^+ ^T cells in 96 wells plate. T cell activation was measured as up-regulation of CD69 after 12 h of culture.

### In vivo activation of naïve CD4^+ ^T cells during malaria blood stage

In order to test the *in vivo *activation of naïve CD4^+ ^T cells during malaria blood stage, adoptive transfer of DO11.10 cells was performed. 1.6 × 10^6 ^naïve DO11.10 CD4^+ ^T cells from transgenic mice were labeled with CFSE (10 μM for 45 min) and transferred (i.v.) into groups of three uninfected or infected Balb/c mice 5 or 10 days after infection (sex and age of donor and recipient mice were matched). Mice were immunized with 2 mg/mouse of OVA emulsified in complete Freund's adjuvant (Sigma) by i.p. injection 24 h after transfer. Proliferation and expression of CD11a, CD62L and intracellular IL-2 was determined in transferred CFSE^+ ^CD4^+ ^T-cells 3 days after immunization by FACS. Control mice received the same volume of adjuvant alone.

### Ethical approval

Experiments performed with mice were approved by the NYU Institutional Animal Care and Use Committee (IACUC).

## Results and Discussion

**Figure 1:**
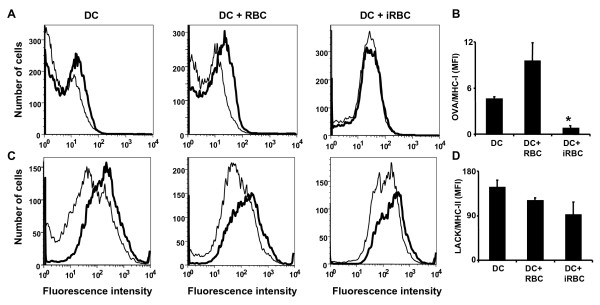
***Plasmodium yoelii*-infected erythrocytes inhibit MHC-I but not MHC-II antigen presentation**. (A-D) DCs differentiated *in vitro *were incubated alone (DC), with uninfected (DC+RBC), *P. yoelii*-infected erythrocytes (DC+iRBC) for 24 h before addition or not of purified OVA (5 mg/ml; A,B) or LACK (50 μg/ml; C,D) proteins for 5 h, followed by stimulation with LPS to increase antigen presentation. Cells were stained with antibodies recognizing (A,B) OVA epitope 257–264 bound to *H-2*^*b *^(antibody 25-D1) or (C,D) LACK epitope 156–173 bound to I-A^d ^(antibody 2C44). (A,C) Representative examples of FACs histograms of DCs incubated with protein OVA (A) or LACK (C) proteins (thick lines) or control DCs (thin lines). (B,D) Results are expressed as the difference in mean fluorescent intensity (MFI) of protein incubated DCs minus control DCs in triplicated determinations. *, significant difference (P < 0.01) in the intensity compared with DCs incubated alone.

DCs present exogenous antigens in the context of MHC class II molecules for the activation of CD4^+ ^T cells. DCs are also able to cross-present exogenous antigens in the context of MHC-I molecules to activate CD8^+ ^T cells. It was determined whether MHC-II and MHC-I antigen presentation was affected during infection with *P. yoelii*. For this purpose, specific antibodies were used, that recognize defined peptide epitopes bound to particular MHC molecules. These antibodies do not recognize the antigen or the MHC molecules alone and can be used to determine the presence of MHC-epitope bound complexes in the surface of antigen presenting cells. To determine MHC-I and MHC-II antigen presentation anti-OVA epitope 257–264/H-2^b ^(25-D1.16) and anti-LACK epitope 156–173/I-A^d ^(2C44) antibodies were used, respectively. Bone-marrow derived DCs were incubated either alone, with uninfected or *P. yoelii*-infected erythrocytes for 24 h before addition of purified OVA or LACK proteins for 5 h, followed by stimulation with LPS to induce antigen presentation. It was observed that incubation with *P. yoelii*-infected erythrocytes inhibits presentation of exogenous antigens on MHC-I (Figures 1A and 1B), but not on MHC-II molecules (Figures 1C and 1D).

**Figure 2:**
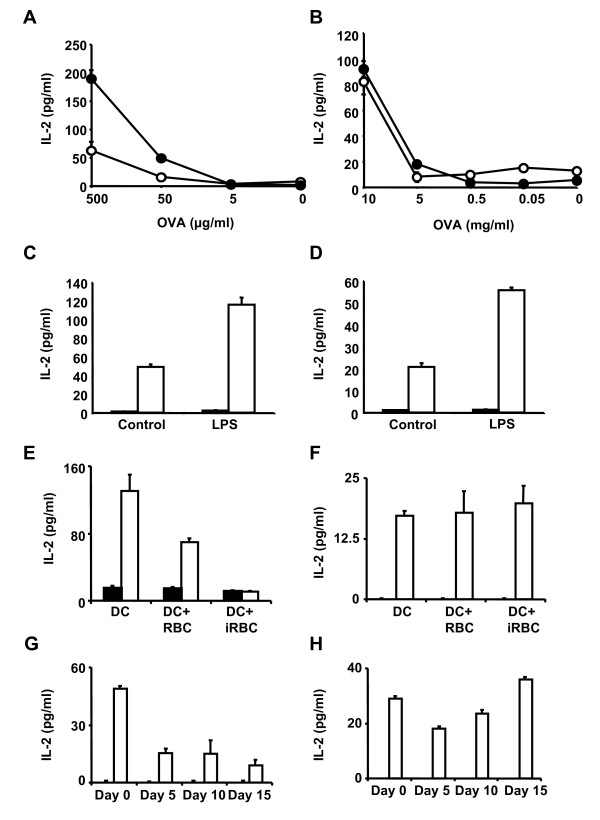
**DCs from *P. yoelii*-infected mice can process exogenous antigen for MHC class II presentation but not for MHC class I**. (A,B) DCs pre-incubated with uninfected (black circles) or *P. yoelii*-infected (white circles) erythrocytes for 24 h, were incubated with OVA at the indicated concentrations for 5 h. DCs were then fixed and incubated with hybridoma B3Z specific for OVA epitope 257–264/*H-2*^*b *^(MHC-I) (A) or hybridoma DO11.10 specific for OVA epitope 323–339/*I-A*^*d *^(MHC-II) (B). (C,D) DCs were incubated with BSA (5 mg/ml; balck bars) as negative control or OVA (5 mg/ml; white bars) for 5 h before addition or not of LPS for 16 h. DCs were then incubated with B3Z (C) or DO11.10 (D) hybridomas for 24 h. (E-H) DCs alone, pre-incubated with uninfected or *P. yoelii*-infected erythrocytes (E,F) or CD11c^+ ^DCs isolated from infected mice at different times during blood-stage infection (G,H) were incubated with OVA 5 mg/ml (white bars) or BSA 5 mg/ml as negative control (black bars) for 5 h before addition of LPS for 16 h. DCs were fixed and incubated with hybridoma B3Z (E,G) or hybridoma DO11.10 (F,H). After 24 h, secretion of IL-2 by hybridomas in the culture medium was detected by ELISA.

The activation of T cell hybridomas that recognize specific OVA peptides in the context of MHC-I and II molecules was used as an alternative method to determine antigen presentation. The T cell hybridomas B3Z [9] and DO.11.10 [10] recognize specific OVA peptides on MHC class I (*H-2*^*b*^) and II (*I-A*^*d*^), respectively. T hybridoma cells are less dependent than primary naive T cells on costimulatory molecules and they can be activated by fixed antigen presenting cells. Recognition of peptide-MHC complexes by T hybridoma cells results in increased IL-2 secretion. DCs were incubated with uninfected or *P. yoelii*-infected erythrocytes for 24 h before addition of purified OVA proteins for 5 h. DCs were then fixed to prevent secretion of cytokines and incubated with the different hybridomas for 24 h. Activation of hybridomas was determined by detection of IL-2 in the incubation medium. It was found that DCs pre-incubated with *P. yoelii*-infected erythrocytes activate the T cell hybridoma recognizing OVA-MHC-II, but the activation of the hybridoma recognizing OVA-MHC-I was significantly reduced. The activation of the hybridomas was dose dependent (Figures 2A and 2B).

Maturation of DCs increases their capacity for antigen presentation, since it increases the expression of MHC molecules on the cell surface [1]. As expected, it was found that the addition of LPS to induce maturation of DCs 5 h after incubation with OVA, resulted in increased activation of both hybridomas recognizing OVA-MHC-I and II (Figures 2C and 2D). The effect of pre-incubating DCs with *P. yoelii*-infected or uninfected erythrocytes in antigen presentation of OVA by MHC-I and MHC-II after inducing maturation with LPS was analysed. It was again observed that *P. yoelii*-infected erythrocytes inhibit the activation of the hybridoma recognizing OVA-MHC-I, but not OVA-MHC-II (Figures 2E and 2F).

The capacity of CD11c^+ ^DCs isolated from *P. yoelii*-infected mice to present antigens by MHC-II was analysed. It was found that that MHC-II antigen presentation is maintained during the course of infection, but MHC-I cross-presentation of exogenous antigens is inhibited throughout the course of the disease (Figures 2G and 2H). These results confirm previous observations [11] that MHC-I antigen presentation is inhibited during *Plasmodium *infections and indicate that DCs are able to process and present exogenous antigens by MHC-II during *P. yoelii *infections.

**Figure 3:**
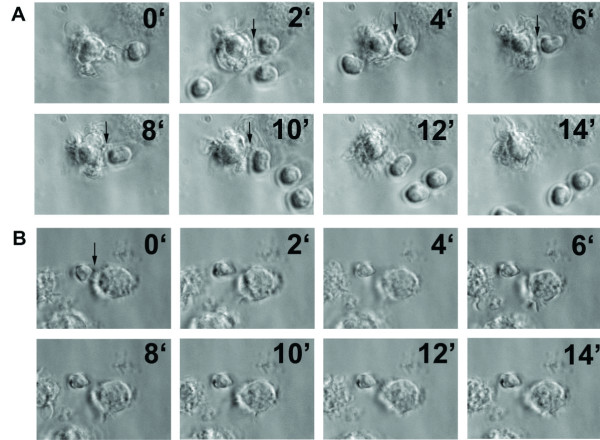
**Time-lapse microscopic analysis of the interaction between DCs and T cells after pre-incubation with *P. yoelii*-infected erythrocytes**. DCs were differentiated *in vitro *and pre-incubated with uninfected (A) or *P. yoelii*-infected (B) erythrocytes before loading with OVA peptide 323–339. Naïve DO11.10 T cells that are specific for this OVA epitope were isolated from transgenic mice and added to DCs. Individual pictures frames from movies (Additional file 1 and Additional file 2) showing DC-T cell interactions. Arrows indicate contacts between DCs and T cells. Time in min is indicated in each frame. Representative results from one of five independent experiments are shown.

The initial encounters between antigen-presenting DCs and specific naïve T cells include directional projection of abundant membrane extensions from the DC toward the naïve T cell and prolonged interactions between both [2]. To analyse whether *Plasmodium *interferes with this process, the interactions between bone marrow derived DCs pre-incubated with infected erythrocytes and naïve CD4^+ ^T cells were first studied using time-lapse video microscopy. DCs were pre-incubated with uninfected or *P. yoelii*-infected erythrocytes and incubated with naïve anti-OVA CD4^+ ^T cells isolated from transgenic mice (DO11.10) recognizing a specific OVA epitope. DCs were loaded with the same peptide epitope (OVA 323–339) before addition of T cells. DCs presenting antigen normally interact with antigen-specific naïve T cells for long periods of time (more than 5 min) with abundant membrane extensions projected in the direction of the T cell [8]. Pre-incubation of DCs with uninfected erythrocytes did not affect DC-T cell interactions, as prolonged interactions with membrane extensions were frequently found in the co-cultures (Figure 3A and Additional file 1). In contrast, pre-incubation with *P. yoelii*-infected erythrocytes inhibits the capacity of DCs to maintain prolonged interactions with membrane extensions with naïve T cells (Figure 3B and Additional file 2). Only short interactions without projection of membrane extensions were observed.

**Figure 4:**
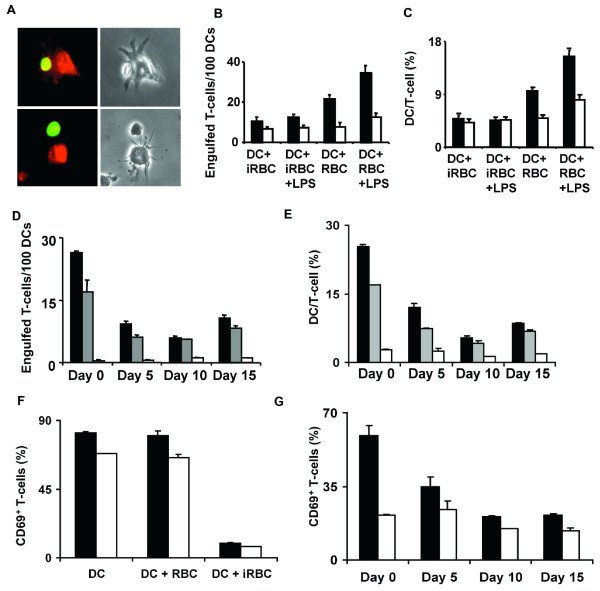
**Impaired ability of DCs to interact with and to prime naïve CD4**^+^**T cells**. (A) Upper left panel shows a T-cell (green) 'engulfed' by a DC (red). Lower left panel shows a T-cell in contact with a DC but not 'engulfed'. Right panels show the same microscopic fields in transmitted light. (A-C) DCs were differentiated *in vitro *and pre-incubated with uninfected (DC+RBC) or *P. yoelii*-infected (DC+iRBC) erythrocytes, followed or not by incubation with LPS. DCs were loaded with OVA peptide 323–339 (black bars) or not (white bars) and incubated for 30 min with naïve DO11.10 T-cells labeled with CFSE (green). (D,E) CD11c^+ ^DCs were isolated from *P. yoelii*-infected mice at different times of infection and incubated alone (white bars), with OVA peptide 323–339 (gray bars), or with LPS and OVA peptide 323–339 (black bars). DCs were incubated for 30 min with naïve DO11.10 T-cells labeled with CFSE (green). For microscopy analysis (A,B,D), DCs were fixed and actin was stained (red), for FACs analysis (C,E), DCs were labeled with cell tracker (red) before incubation with T-cells. (B,D) Number of 'engulfed' T-cells per 100 DCs analysed by microscopy. (C,E) Percentage of DC-T-cell conjugates analysed by FACS. (F,G) Up-regulation of CD69 in naïve CD4^+ ^T-cells after a12 h incubation with OVA peptide 323–339-loaded unstimulated (white bars) or LPS-stimulated DCs (black bars). (F) DCs were differentiated *in vitro *and pre-incubated or not with uninfected or *P. yoelii*-infected erythrocytes. (G) DCs were isolated from infected mice at different times during blood-stage infection. Results are expressed as mean ± SD of triplicate samples. FACs analysis for (F) and (G) data is shown in Additional File 3.

To perform a quantitave analysis of this phenomenon, the formation of stable conjugates between antigen-presenting DCs and specific T cells was observed. Each cell type population was labeled with a different fluorescent dye to allow the determination of dual positives for both labels that correspond to stable conjugates [8]. After a 30 min co-incubation of equal numbers of DCs presenting the ovalbumin epitope and the specific naïve anti-OVA CD4^+ ^T cells formed stable conjugates where DCs 'engulf' T cells (Figure 4A, upper panels), which are different from loose contacts between these cells (Figure 4A, lower panel).

Quantification of the number of dual positives by fluorescence microscopy revealed that DC-T cell conjugates are formed when control DCs loaded with the specific peptide epitope are incubated with specific T cells and the number of conjugates is increased when DCs had been activated by addition of LPS. Pre-incubation of DCs with *P. yoelii*-infected erythrocytes significantly inhibited the formation of stable DC-T cell conjugates (Figure 4B). Analysis of double positives for each fluorescent label by FACs provided similar results (Figure 4C). Addition of LPS as a maturation signal increased the number of DC-T cell conjugates in the control DCs pre-incubated with uninfected erythrocytes, but did not improve DC-T cell interactions impaired by *P. yoelii*-infected erythrocytes (Figures 4B and 4C).

When a similar analysis was performed using CD11c^+ ^DCs isolated from *P. yoelii*-infected animals at different times after infection, we also found a significant decrease in the numbers of DC-T cell stable conjugates compared to uninfected mice (day 0). Even if lower, detectable levels of stable conjugates formed by DCs from infected mice and specific T cells were found by microscopy and FACs (Figures 4D and 4E), suggesting that DC-T cell interactions are impaired but not completely inhibited by *P. yoelii *infection. These levels were minimally increased by addition of LPS to DCs (Figures 4D and 4E).

To determine the level of T cell activation in the co-cultures of DC and T cells, DCs were activated by addition of LPS and the surface expression of the early activation marker CD69 in T cells were analysed. In the co-cultures of DCs incubated with *P. yoelii*-infected erythrocytes or isolated from infected mice, the level of T cells expressing CD69 was greatly decreased compared to co-cultures with control DCs (Figures 4F and 4G).

**Figure 5:**
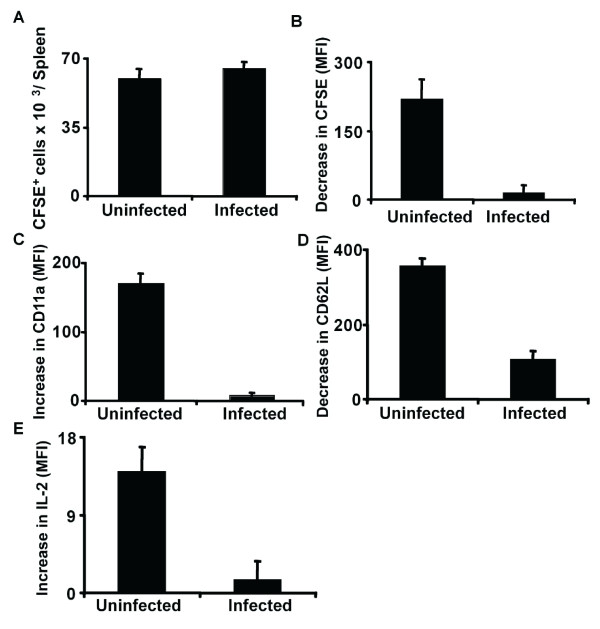
**Activation of naïve CD4^+ ^T-cells is impaired during late *Plasmodium *blood stage infection**. DO11.10 naïve CD4^+ ^T-cells isolated from the spleens of transgenic mice were labeled with CFSE and transferred into uninfected or *P. yoelii*-infected mice (10 days after infection). Mice were immunized or not with OVA 24 h after transfer of T-cells. Three days after immunization, transferred CD4^+ ^T-cells from spleens of recipient mice were analysed by FACs for (A) total number in the spleen, (B) proliferation (determined as decrease in CFSE fluorescence), (C) up-regulation of CD11a surface expression, (D) down-regulation of CD62L surface expression, and (E) increase in intracellular IL-2 production. Results are expressed as the difference between values obtained with non-immunized versus OVA-immunized mice. Results are expressed as mean ± SD of triplicate samples. FACs analysis with the gates for transferred cells are shown in Additional file 4.

To study the activation of CD4^+ ^T-cells during *P. yoelii *infection *in vivo*, naïve CD4^+ ^T cells were transferred from DO11.10 transgenic mice that are specific for the OVA epitope 323–339 into *P. yoelii*-infected (day 10 p.i.) or uninfected mice. Mice were immunized with OVA 24 h after transfer and CD4^+ ^T-cell activation was measured 72 h after OVA injection. Naïve CD4^+ ^T-cells were fluorescently labeled before transfer to allow identification. The number of transferred CD4^+ ^T-cells in the spleen after 72 h was similar in uninfected and infected animals (Figure 5A), but after immunization with antigen, infected mice primed transferred naïve CD4^+ ^T-cells with lower efficiency than uninfected mice. T-cell activation was lower in infected mice as determined by proliferation (decrease in CFSE labeling, Figure 5B), up-regulation of CD11a (Figure 5C), down-regulation of CD62L (Figure 5D) and increased intracellular IL-2 (Figure 5E). The effect of transferring naïve CD4^+ ^T cells from DO11.10 transgenic mice into *P. yoelii*-infected mice earlier in infection (day 5), was also analysed. Under these conditions, only proliferation (decrease in CFSE labeling) and expression of CD11a were significantly decreased in infected animals. No significant changes were found in IL-2 secretion and expression of CD62L.

The capacity of DCs to provide adequate antigen presentation to T cells in the context of *P. yoelii *infection was analysed in detail. The results suggest that during *P. yoelii *infections DCs maintain the ability to process and present exogenous antigens by forming the MHC-II-epitope complex in the surface of DC, however, as shown before for *P. berghei *[11], impaired cross-presentation of exogenous antigens by MHC-I was found.

Efficient antigen presentation is not sufficient for the activation of naïve CD4^+ ^and CD8^+ ^T cells, which also requires co-stimulatory signals from DCs and specific cytokines [12]. The interactions of naïve T cells with mature priming-inducing DCs are more stable than the contacts with resting tolerance-inducing DCs. It has been proposed that stable DC-T cell interactions participate in the induction of antigen-specific T cell activation through the delivery of an activatory signal by the DCs. In contrast, unstable contacts with resting DCs might induce short-term activation and proliferation signals in T cells [2], which may explain the lack of T cell activation that we observed during late malaria infection. In the absence of mature DCs, serial unstable contacts between T cells and resting DCs would result in T cell clonal deletion [2], a phenomenon that has also been observed during malaria infections, where there is specific deletion of T cells recognizing *Plasmodium *antigens [13].

Maturation of DCs increases the duration of DC-T cell interactions and allows the formation of a complex net of membrane extensions in which DCs entrap T cells [8]. The capacity of DCs to establish these effective interactions with naïve CD4^+ ^T cells was found to be inhibited during infection. As actin-mediated reorganization of DC morphology is required to form the strong, long-lasting interactions with T cells [8], it is possible that the parasite may interfere with the cytoskeleton of the DCs. In fact, numerous genes in the DC cytoskeleton are modulated during *P. yoelii *infection [14].

Previous studies have shown that DCs can process and present antigens associated with MHC class II during blood stage infections, suggesting that efficient activation of CD4^+ ^T cells could take place during murine infection [15], however, defective activation and specific depletion of CD4^+ ^T cells recognizing *Plasmodium *antigens is observed during murine malaria infections [13,16]. Suppression of OVA-specific CD4^+ ^T cell proliferation has also been observed in *P. chaubaudi*-infected mice, but in this model inhibition was evident since early infection [17]. Furthermore, these authors also found that DC and CD4^+ ^T cell interactions are inhibited by *P. chaubaudi *infection *in vitro *and *in vivo*, while antigen presentation by MHC-II is not affected [18].

Recently, two subpopulations of CD11c^+ ^DCs with differential abilities to activate antigen-specific T cells have been identified in *P. chaubaudi *infected mice [19], suggesting that there may be a balance of opposing forces on the host response. Since the interactions between DCs and T cells are decreased, but are still detectable, it is possible that we have observed the sum of different effects contributed by different subpopulations of DCs. It is currently believed that tolerance of DCs, induced by exposure to TLR ligands is induced during malaria infection [20]. In this context, it is likely that tolerized DCs that are found increasingly during late infection would have impaired interactions with T cells leading to decreased activation.

## Conclusion

In this work, it was observed that DCs isolated from infected mice at different times after infection are not able to establish strong interactions and prime naïve CD4^+ ^T cells. Protection generated by *Plasmodium *infections against blood-stage infection is mediated by helper and effector functions of CD4^+ ^T cells [21]. However, this T cell response does not induce complete protection or long-term immunity, suggesting that T cell activation or maintenance is impaired [22]. These results indicate that activation of naïve CD4^+ ^T cells by DCs is impaired during late malaria blood-stage infection in mice.

## Abbreviations

DCs: Dendritic cells; OVA: ovalbumin; CFSE: 5-(and-6)-carboxyfluorescein diacetate, succinimidyl ester.

## Competing interests

The authors declare that they have no competing interests.

## Authors' contributions

CO, KAW and AR conceived the study, participated in its design and coordination and wrote the manuscript. All authors read and approved the final manuscript.

## Supplementary Material

Additional file 1 DCs were differentiated *in vitro *and pre-incubated with control uninfected erythrocytes before loading with OVA peptide 323–339. Naïve DO11.10 T cells that are specific for this OVA epitope were isolated from transgenic mice and added to DCs. Movie shows prolonged interaction between DC and T cell. Click here for file

Additional file 2 DCs were differentiated *in vitro *and pre-incubated with *P. yoelii*-infected erythrocytes before loading with OVA peptide 323–339. Naïve DO11.10 T cells that are specific for this OVA epitope were isolated from transgenic mice and added to DCs. Movie shows defective interaction between DC and T cell. Click here for file

Additional file 3 DCs were differentiated *in vitro *and pre-incubated or not with uninfected or *P. yoelii*-infected erythrocytes (upper panels) or isolated from infected mice at different times during blood-stage infection (lower panels). Naïve DO11.10 CD4^+ ^T-cells were incubated for 12 h with LPS-stimulated DCs loaded with OVA peptide 323–339. Up-regulation of CD69 was measured in CD4^+ ^T cells. This is a representative example of triplicated sample FACS analysis. Click here for file

Additional file 4 DO11.10 naïve CD4^+ ^T-cells isolated from the spleens of transgenic mice and transferred into uninfected or *P. yoelii*-infected mice (10 days after infection). Mice were immunized or not with OVA 24 h after transfer of T-cells. Three days after immunization, transferred CD4^+ ^T-cells from spleens of recipient mice were analysed by FACs. As control for FACs analysis mice infected or not that were not transferred with T cells were used. Transferred cells were identified using an antibody specific for DO11.10 TCR. Click here for file
