# Supplementary material for: Interactions between dendritic cells and CD4+ T cells during Plasmodium infection
Source: Malar J. 2008 May 21;7:88. doi: 10.1186/1475-2875-7-88 (PMC2423365; doi:10.1186/1475-2875-7-88)
Supplement: Additional file 1 — DCs were differentiated in vitro and pre-incubated with control uninfected erythrocytes before loading with OVA peptide 323–339. Naïve DO11.10 T cells that are specific for this OVA epitope were isolated from transgenic mice and added to DCs. Movie shows prolonged interaction between DC and T cell. [file 1475-2875-7-88-S1.ppt]

## Slide 1
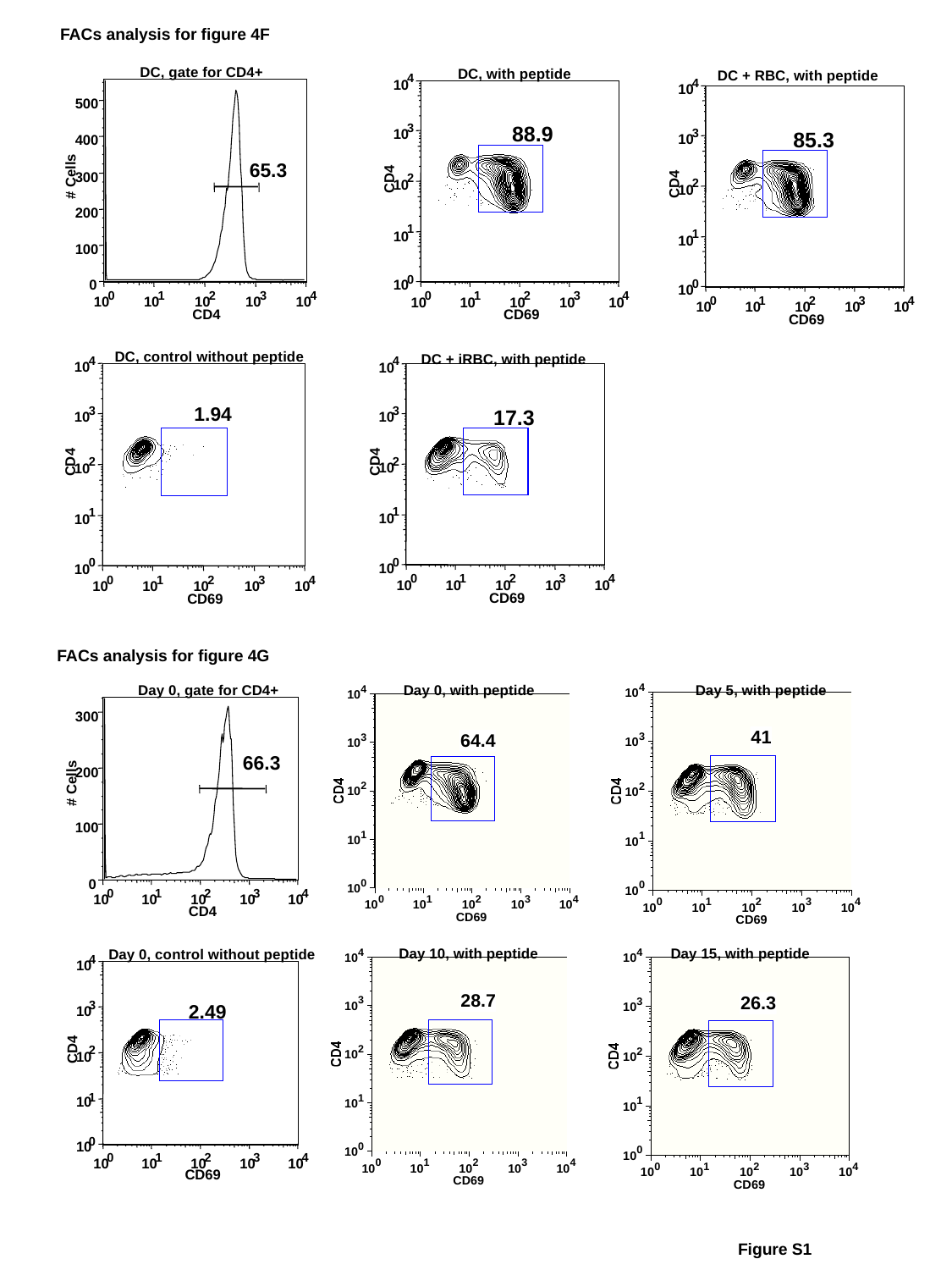

FACs analysis for figure 4F
DC, gate for CD4+
DC, with peptide
DC + RBC, with peptide
4
10
3
10
2
10
1
10
0
10
CD4
0
1
2
3
4
10
10
10
10
10
CD69
88.9
4
10
3
10
2
10
1
10
0
10
CD4
0
1
2
3
4
10
10
10
10
10
CD69
85.3
500
400
300
200
100
0
# Cells
0
1
2
3
4
10
10
10
10
10
CD4
65.3
DC, control without peptide
DC + iRBC, with peptide
4
10
3
10
2
10
1
10
0
10
CD4
0
1
2
3
4
10
10
10
10
10
CD69
1.94
4
10
3
10
2
10
1
10
0
10
CD4
0
1
2
3
4
10
10
10
10
10
CD69
17.3
FACs analysis for figure 4G
Day 0, gate for CD4+
Day 0, with peptide
Day 5, with peptide
300
200
100
0
# Cells
0
1
2
3
4
10
10
10
10
10
CD4
66.3
Day 10, with peptide
Day 15, with peptide
Day 0, control without peptide
4
10
3
10
2
10
1
10
0
10
CD4
0
1
2
3
4
10
10
10
10
10
CD69
2.49
Figure S1
