# Supplementary material for: Interactions between dendritic cells and CD4+ T cells during Plasmodium infection
Source: Malar J. 2008 May 21;7:88. doi: 10.1186/1475-2875-7-88 (PMC2423365; doi:10.1186/1475-2875-7-88)
Supplement: Additional file 2 — DCs were differentiated in vitro and pre-incubated with P. yoelii-infected erythrocytes before loading with OVA peptide 323–339. Naïve DO11.10 T cells that are specific for this OVA epitope were isolated from transgenic mice and added to DCs. Movie shows defective interaction between DC and T cell. [file 1475-2875-7-88-S2.ppt]

## Slide 1
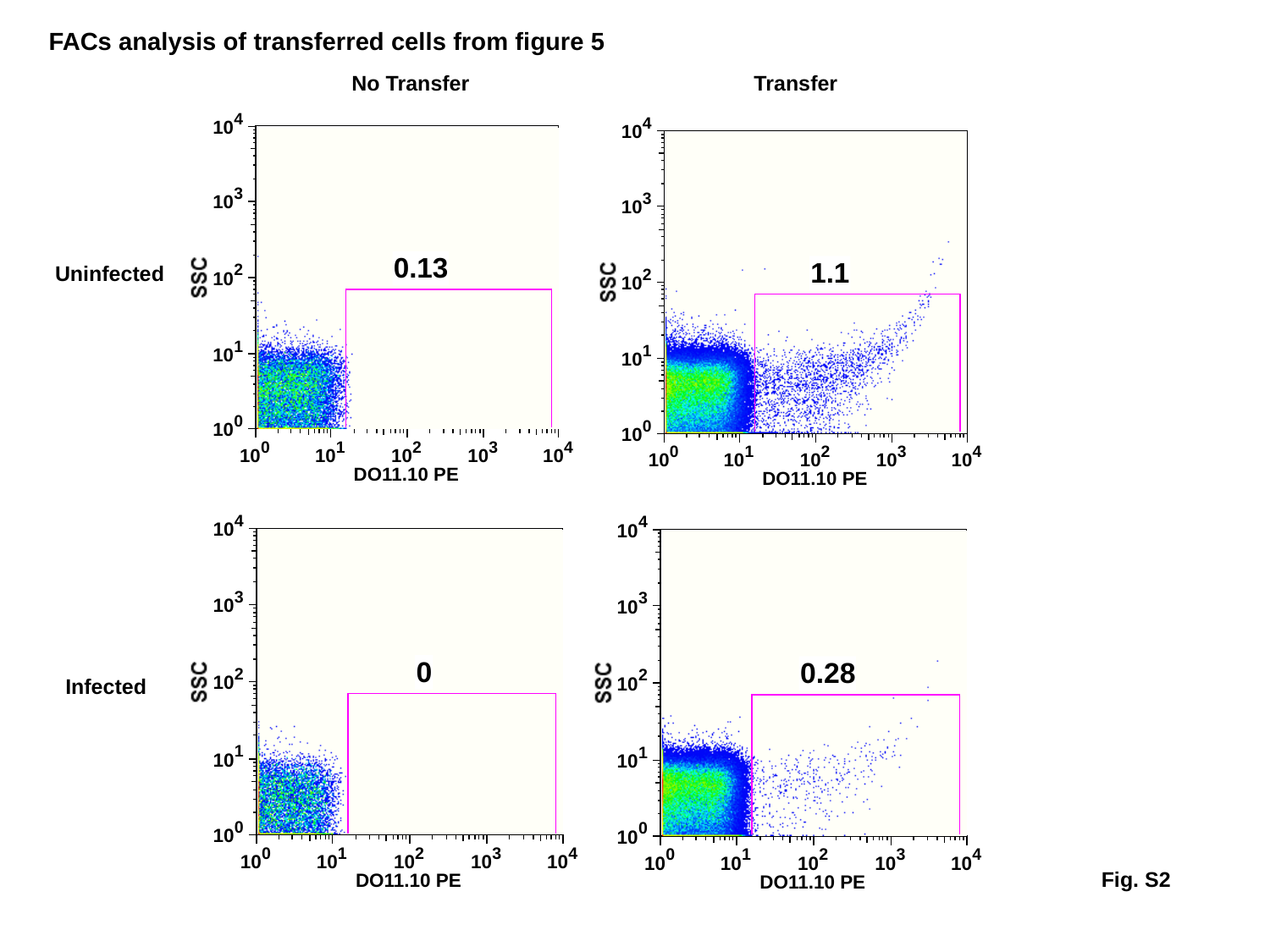

FACs analysis of transferred cells from figure 5
No Transfer
Transfer
Uninfected
Infected
Fig. S2
